# Key issues in recruitment for minority aging research in humanitarian settings

**DOI:** 10.1002/alz.090987

**Published:** 2025-01-09

**Authors:** Rosa P. Pirela‐Mavarez, Chun Xu, Joseph D. Terwilliger, Noe Garza, Roberto Reyes, Omar Oropeza, Eron Grant Manusov, Cristian A. Maestre, Ney Alliey‐Rodriguez, Silvia Mejia‐Arango, Jesus D Melgarejo, Cynthia De La Garza‐ Parker, Gabriel A. de Erausquin, Mario Gil, Romeo Escobar, Neela K. Patel, Sudha Seshadri, Gladys E. Maestre

**Affiliations:** ^1^ The University of Texas Rio Grande Valley School of Medicine, Harlingen, TX USA; ^2^ South Texas Alzheimer’s Disease Research Center, Harlingen, TX USA; ^3^ RGV Alzheimer’s Center (AD‐RCMAR), Brownsville, TX USA; ^4^ Laboratory of Neuroscience, University of Zulia, Maracaibo, Zulia Venezuela (Bolivarian Republic of); ^5^ Institute of Neuroscience at the University of Texas Rio Grande Valley, Harlingen, TX USA; ^6^ The University of Texas Rio Grande Valley, Brownsville, TX USA; ^7^ Division of Medical Genetics, New York State Psychiatric Institute, New York, NY USA; ^8^ Departments of Psychiatry and Genetics & Development, New York, NY USA; ^9^ Sergievsky Center & Department of Epidemiology, Columbia University Medical Center, New York, NY USA; ^10^ Division of Public Health Genomics, National Institute for Health and Welfare, Helsinki Finland; ^11^ University of Texas Rio Grande Valley, Brownsville, TX USA; ^12^ The University of Texas Health Science Center at San Antonio, San Antonio, TX USA; ^13^ South Texas Alzheimer’s Disease Research Center, San Antonio, TX USA; ^14^ Glenn Biggs Institute for Alzheimer’s & Neurodegenerative Diseases, University of Texas Health Science Center, San Antonio, TX USA; ^15^ The University of Texas Rio Grande Valley, Edinburg, TX USA

## Abstract

**Background:**

The increasing number of natural disasters and wars will result in complex emergencies affecting how individuals age. Additionally, the continued complex crises in certain areas of the globe exacerbates risks for chronic health conditions and poverty. Implementing ethical and community‐engaged research in these complex humanitarian settings requires tailored approaches. The South Texas Alzheimer’s Disease Research Center and the Rio Grande Valley Alzheimer’s Disease Resource Center for Minority Aging Research have adopted the humanitarian lens perspective deployed in the Maracaibo Aging Study in Venezuela to promote the recruitment and retention of older adults and their care partners in the Texas/Mexico border. Our objective is to identify and share key issues in recruitment for aging research in a complex humanitarian setting.

**Method:**

Listening sessions, focus groups, and interviews were carried out to understand barriers, facilitators, beliefs, and preferences of older adults, care partners of people living with dementia, promotoras/community health workers, family physicians, and community‐based organizations to understand their expectations better. Elements of the humanitarian framework and capacity building were informed by the qualitative data and guidance from a senior coalition.

**Result:**

Located in the Texas/Mexico border, the Rio Grande Valley constitutes one of the most impoverished areas in the nation. Competing needs, lack of incentives for participation in research, and fear associated with increasing health care costs were the most frequently cited barriers among residents. We decided to implement several aspects of humanitarian work that were related to efficient research and as a response to community expectations. Advocacy for integrating services into primary and community care was supported through staff training, task‐sharing, and establishing referral and follow‐up mechanisms. Diversifying the research workforce with bilingual and multicultural junior investigators and promotoras further addressed the cultural fluidity. As a result, a research registry of over 400 individuals was built, and more than 400 have participated in different aging studies.

**Conclusion:**

Humanitarian settings in high‐income and low‐and middle‐income countries share many implementation needs, including scaling up efforts and tackling multiple challenges simultaneously. Community‐engaged aging research in humanitarian settings requires consideration and assessment of evidence‐based planning and execution and impact evaluation.

